# Effect of a Graduated Walking Program on the Severity of Obstructive Sleep Apnea Syndrome. A Randomized Clinical Trial

**DOI:** 10.3390/ijerph17176334

**Published:** 2020-08-31

**Authors:** Antonio Jurado-García, Guillermo Molina-Recio, Nuria Feu-Collado, Ana Palomares-Muriana, Adela María Gómez-González, Francisca Lourdes Márquez-Pérez, Bernabé Jurado-Gamez

**Affiliations:** 1 Department of Physiotherapy, San Juan de Dios Hospital Cordoba, 14012 Cordoba, Spain; ajuradogarcia@outlook.es; 2 Department of Nursing, Faculty of Medicine and Nursing, University of Cordoba, 14004 Cordoba, Spain; 3 Maimonides Biomedical Research Institute of Cordoba (IMIBIC), Pneumology Department, Reina Sofia University Hospital, University of Cordoba, 14004 Cordoba, Spain; nurifeco@yahoo.es (N.F.-C.); anamerypm@hotmail.com (A.P.-M.); bjgamez@uco.es (B.J.-G.); 4 Cardiopulmonary Rehabilitation Department, Virgen de la Victoria University Hospital, 29010 Malaga, Spain; adelareha@gmail.com; 5 Pneumology Department, University Hospital of Badajoz, 06005 Badajoz, Spain; paquilourdes.marquez@gmail.com

**Keywords:** sleep apnea, cardiovascular diseases, exercise and pulmonary rehabilitation, physical activity

## Abstract

Background: Obstructive sleep apnea syndrome (OSAS) is a common disease. The objective of this research was to determine the effectiveness of a graduated walking program in reducing the apnea–hypopnea index number in patients with obstructive sleep apnea syndrome (OSAS). Methods: A randomized controlled clinical trial with a two-arm parallel in three tertiary hospitals was carried out with seventy sedentary patients with moderate to severe OSAS. Twenty-nine subjects in each arm were analyzed by protocol. The control group received usual care, while usual care and an exercise program based on progressive walks without direct supervision for 6 months were offered to the intervention group. Results: The apnea–hypopnea index decreased by six points in the intervention group, and improvements in oxygen desaturation index, total cholesterol, and Low-Density Lipoprotein of Cholesterol (LDL-c) were observed. A higher decrease in sleep apnea–hypopnea index (45 ± 20.6 vs. 34 ± 26.3/h; *p* = 0.002) was found in patients with severe vs. moderate OSAS, as well as in oxygen desaturation index from baseline values (43.3 vs. 34.3/h; *p* = 0.046). Besides, High-Density Lipoprotein of Cholesterol (HDL-c) values showed a higher increase in the intervention group (45.3 vs. 49.5 mg/dL; *p* = 0.009) and also, a higher decrease in LDL-c was found in this group (141.2 vs. 127.5 mg/dL; *p* = 0.038). Conclusion: A home physical exercise program is a useful and viable therapeutic measure for the management of OSAS.

## 1. Introduction

Obstructive sleep apnea syndrome (OSAS) is a common disease with a prevalence of 19% in men and 14.9% in women in Spain [1]. The OSAS severity is determined by the number of respiratory events during sleep that result in impaired sleep quality, unrefreshing sleep, and excessive daytime sleepiness [2]. These aspects negatively affect areas of occupational health [3], lead to higher levels of anxiety and depression compared to individuals without OSAS, and are related to decreased physical activity and sedentary lifestyles, which, in turn, hinders the clinical management of this disease. Besides, sleep disorders trigger mechanisms associated with endothelial dysfunction [4] and increased risk of vascular events and mortality [5].

The treatment of choice for OSAS is continuous positive airway pressure (CPAP), mandibular advancement devices, and general therapeutic measures [2,6]. These general measures are applicable in all stages of the disease and constitute the only treatment in individuals presenting with mild to moderate OSAS without cardiovascular risk factors [6]. Obesity is potentially modifiable and is one of the most important risk factors in the development of OSAS and stands out among these general measures [7,8]. Observational studies have reported that physical exercise may reduce the prevalence [7,9] and the incidence of sleep-disordered breathing [10], as well as the sleep apnea–hypopnea index (AHI). However, one limitation of these studies is their small sample size [11,12]. In addition, there are no unsupervised physical exercise programs easily performed by all patients and that, in this context, are effective in reducing the severity of OSAS. Moreover, physical activity has a multifactorial component that favors variability across different populations [13].

The main objective of the study was to determine the effectiveness of a progressive walking program in reducing the number of sleep-disordered breathing events in moderate to severe OSAS patients. The secondary objective was to assess the impact of daytime sleepiness on activities of daily living of patients using the Functional Outcomes of Sleep Questionnaire [14], as well as on lipid metabolism.

## 2. Materials and Methods

### 2.1. Study Design

**Figure 1:**
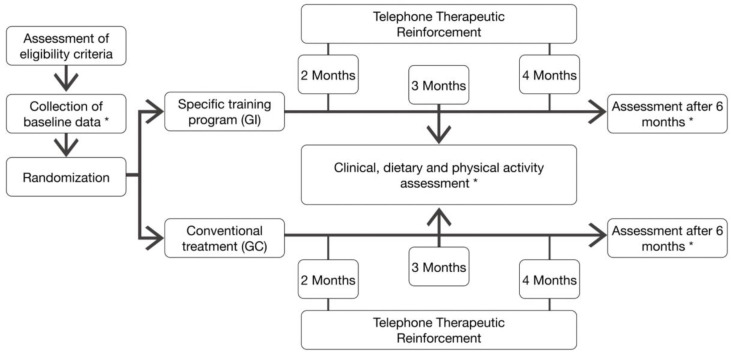
Study Design. * Data collected at baseline and 6 months of intervention: (1) Anthropometric data (Body Mass Index, Neck circumference, Waist-to-hip ratio); (2) Physical activity (steps/day); (3) Questionnaires (Epworth Sleepiness Scale, Functional Outcomes of Sleep Questionnaire]; (4) Sleep-disordered breathing detected through polygraphy (Just for baseline and six months after intervention) (Apneas–Hypopneas Index, SpO2 mean (%), registered time spent with SpO2 < 90% (%) and Oxygen desaturation index); (5) Biochemical parameters (Urea, Creatinine, Alanine aminotransferase, Aspartate aminotransferase, Blood glucose, Total Cholesterol, High-density lipoprotein cholesterol, Low-density lipoprotein cholesterol and Triglycerides).

A randomized controlled clinical trial with a two-arm parallel design was performed in accordance with the CONSORT statement for non-pharmacological trials [15]. Clinical trial registration number: NCT03997877. The study design is shown in Figure 1.

#### 2.1.1. Population

The sample was selected from the 3 University Hospitals participating in the study, from May 2017 to March 2019. Patients with a sedentary lifestyle and suspected sleep-disordered breathing (i.e., the eligible population) were recruited. After an initial examination, a sleep study was requested in order to evaluate the diagnosis and severity of OSAS. The sleep study and data collection were coordinated and conducted at the Reina Sofia University Hospital of Cordoba, Spain. Written informed consent was obtained from the patients, and the study was approved by the Research Ethics Committee of Cordoba (code: SAH-2014, ref.2711).

Data were gathered by one of the authors in collaboration with research coordinators and were analyzed by two of the authors.

#### 2.1.2. Subject Selection

The inclusion criteria were: (1) aged between 18 and 65 years old; (2) low levels of physical activity, defined as obtaining a physical activity index less than 51 on the Yale Physical Activity Survey [16]; (3) an AHI of between 15 and 30/h, an Epworth score less than 12 without vascular risk factors, or an AHI greater than 30/h with CPAP refusal; (4) signing the informed consent form.

Participants were excluded for the following reasons due to advanced chronic illness that precludes following a physical exercise program: (1) impairment of the locomotor system; (2) respiratory failure caused by chronic cardiopulmonary disease; (3) severe psychiatric illness preventing the understanding of and/or compliance with instructions.

#### 2.1.3. Sample Size

For a difference in AHI means of −7.22 (±2.79) events/hour in the experimental group, compared to 0.13 (±1.88) in the control group, representing an effect size (Cohen’s *d*) of −0.945 [17], with the probability of making a type I error being 0.05 for two-tailed tests and a power (1-ß) of 85%, a total size of 44 individuals was estimated to be required—22 for the experimental group and 22 for the control group. To mitigate the possible effect of lost data, this was increased by 10%, resulting in a final minimum sample size of 48 individuals, 24 for each group.

#### 2.1.4. Intervention

**Figure 2:**
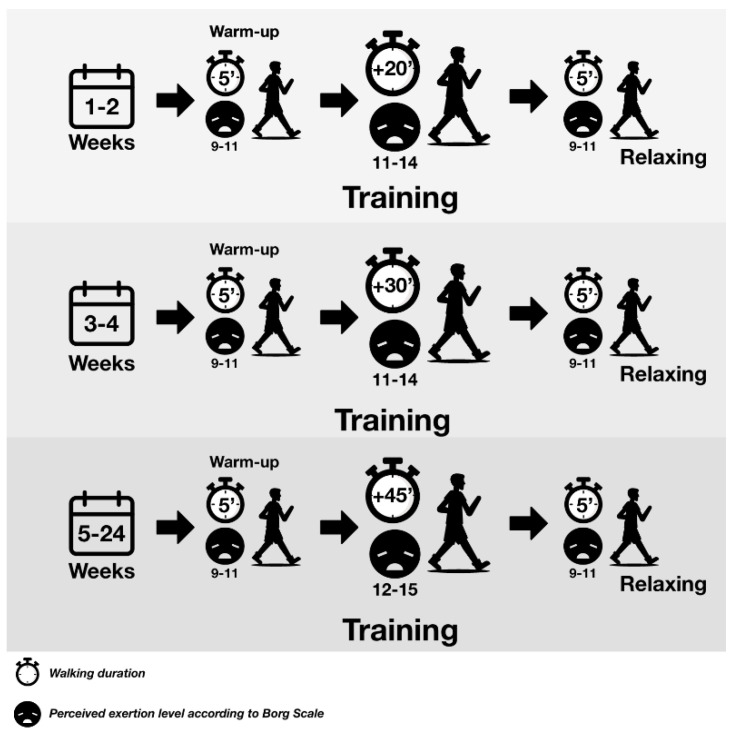
The walking program carried out at least five days a week according to the degree of perceived exertion by the Borg scale.

The control group received general therapeutic measures and regular physical activity monitored with a pedometer was recommended. In addition to these general measures, the intervention group was encouraged in the implementation of an exercise program based on progressive walks without direct supervision for 6 months. The subjects assigned to this group were assessed by a physiotherapist who explained the training program to them (Figure 2). In both groups, physical activity was recorded in a walking diary. Both groups were followed up via telephone in the second and fourth months and were clinically assessed in the third and sixth months (Figure 1).

#### 2.1.5. Bias Control

- Randomization. A random number table was prepared by a statistician who did not participate in the study, using the sampling module of the software Epidat 4.1 (Department of Sanidade, Xunta de Galicia, Galicia, Spain). The ratio of subjects assigned to each group was 1:1.
- The measures for keeping the research team blinded were as follows. The random numbers were kept in sealed envelopes by the Sleep Unit staff who did not participate in the assessment of the outcomes. The subjects were always monitored by the same researcher and no information was given to them about the therapeutic arm they were assigned to. After randomization, the subjects assigned to the interventional group were referred to physiotherapist, while the subjects of the control group continued with their usual care. Information on the therapeutic arm assigned to the patient, the clinical trial nature, or the research hypothesis was avoided. Furthermore, although the physiotherapist knew only the patients in the experimental group, he did not intervene in any way in measuring the effect of the intervention. Finally, the primary outcome (change in the AHI) is an objective determination (sleep test) that cannot be modified by the information given to the patient. For the follow-up, the telephone calls were made by a blinded researcher (she did not know who was assigned to the control or intervention group). They were only performed to reinforce the patient’s continuity in the study and to resolve any doubts.
- Control for healthy habits. All individuals were advised to maintain a stable sleep habit for at least 7 h at night, to refrain from drinking alcohol and taking muscle relaxants during the evening, and to maintain the dietary pattern recommended by the American Heart Association [18]. These recommendations and an explanatory leaflet were provided.

### 2.2. Measures

The data were collected from the patients’ medical records, from their physical examinations, and from the assessment of their degree of physical activity, quantified as steps per day. All the measures, excepting polygraphy, were collected at baseline and 3 and 6 months. Polygraphy was performed at baseline and the end of the study (after six months of intervention).

#### 2.2.1. Clinical Characteristics

Assessment of sleepiness: the degree of daytime sleepiness was assessed using the validated Spanish version of the Epworth Sleepiness Scale [19], while the impact of excessive daytime sleepiness on activities of daily living was assessed using the Spanish version of the Functional Outcomes of Sleep Questionnaire [14].

#### 2.2.2. Cardiorespiratory Polygraphy

The home test was agreed upon by the research teams of the three participating hospitals in accordance with the protocol conducted in Spain [2,20]. In short, airflow was studied using thermistors and pressure signals. Snoring and both thoracic and abdominal effort were recorded using impedance belts, and heart rate and peripheral oxygen saturation (SpO_2_) values were recorded using pulse oximetry. Apnea was defined as a significant drop (>90%) in the airflow signal lasting ≥10 s. Hypopnea was defined as an evident decrease in airflow signal amplitude (>30% and <90%) lasting more than 10 s, accompanied by a ≥4% drop in SpO_2_. The following respiratory variables were analyzed: the AHI or the sum of the number of apneas plus the number of hypopneas per recording hour, mean SpO_2_, and oxygen desaturation index (number of decreases in SpO_2_ ≥ 3%/h) and T90% (recording time spent with a SpO_2_ of <90%). An apnea–hypopnea index ≥5/h was considered diagnosis of OSAS. Subjects were classified as having mild (AHI > 5/h and < 15/h), moderate (AHI > 15/h and < 30/h), or severe (AHI > 30/h) OSAS [2]. All tests were analyzed manually and always by the same researcher, in accordance with the recommended guidelines [2,21].

#### 2.2.3. Physical Activity

In both groups, physical activity was measured using a pedometer (Geonaute ONstep400, Oxylane, Villeneuve-d’Ascq, France) with the ability to store daily data over a week.

### 2.3. Outcomes

The primary outcome was the influence of the physical exercise program on AHI at the end of the follow-up period.

The variables to be controlled for were the following: (1) SpO_2_ values: SpO_2_ during wakefulness (%), mean SpO_2_ (%), oxygen desaturation index, and T90% or amount of recording time spent with a SpO_2_ of <90%; (2) Anthropometric measures: age, sex, body mass index, neck circumference (cm), and waist-to-hip ratio; (3) Physical activity: number of steps per day recorded by the pedometer; (4) Biochemical profile.

### 2.4. Data Analysis

The data were described using means, standard deviations, and minimum and maximum values for quantitative variables, whereas frequencies and percentages were used for qualitative variables. At the beginning of the study, for the comparison of means, Student’s t-test for independent samples or the Mann–Whitney U-test were used. For the comparison of means at the beginning of the study (baseline) and means at the end, Student’s *t*-test for paired samples or Wilcoxon’s signed-rank test were used. The statistical significance threshold for all values was set at *p* < 0.05.

## 3. Results

**Figure 3:**
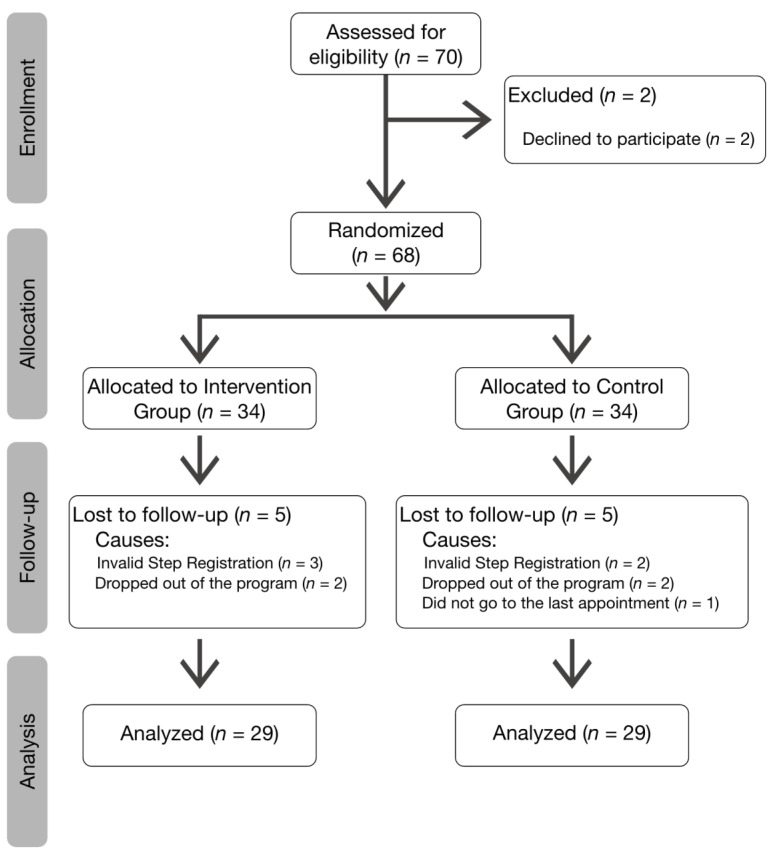
CONSORT flow diagram.

During the recruitment period, the eligible population was 70 subjects. Figure 3 shows the flow of patients. Over the course of the study, five subjects were excluded from each group, resulting in 29 patients in each arm.

The sample had a mean age of 51 ± 8.2 years, a body mass index of 32 ± 4.7 kg/m^2^, an AHI of 28 ± 15.6/h, and an Epworth scale score of 9 ± 4.4. The baseline data for each group were homogeneous, except for blood glucose and oxygen desaturation index (Table 1).

Table 2 shows the changes in the experimental and control groups in baseline variables and after 6 months. In both groups, body mass index and neck circumference decreased. The AHI decreased by six events/h points in the experimental group and one event/h in the control group, although without reaching statistical significance. In this experimental group, improvements were also observed in oxygen desaturation index, total cholesterol, and LDL-c, with a tendency towards statistical significance and a positive effect on excessive daytime sleepiness and the Functional Outcomes of Sleep Questionnaire. The daily distance reached at 6 months was higher by 602.2 steps per day in the experimental group (*p* < 0.005).

**Figure 4:**
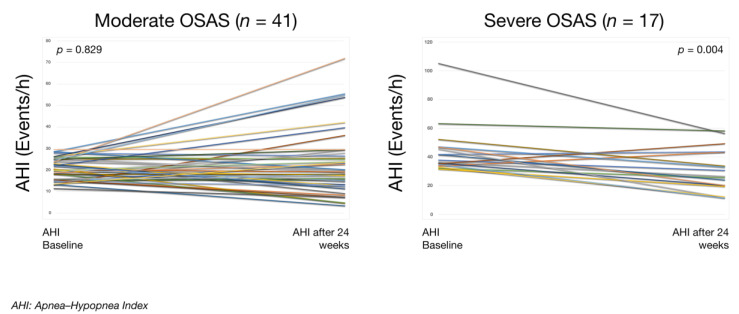
Apnea–hypopnea index before (baseline) and after a physical exercise program (intervention) in patients with moderate vs. severe OSAS. Each line of different colour represents the individual evolution of AHI for each participant. Within-groups comparison using Wilcoxon’s test for asymmetry distribution. The *p*-value represents the level of statistical significance of the Student paired t-test for Moderate OSAS and Wilcoxon’s signed-rank test for Severe OSAS.

When both groups were classified in moderate OSAS (*n* = 41) versus severe OSAS (*n* = 17), as shown in Table 3 and Figure 4, a significant decrease in AHI, oxygen desaturation index, and changes in lipid metabolism were also observed.

## 4. Discussion

In patients with a sedentary lifestyle with moderate–severe OSAS, a 6-month physical activity program did not significantly decrease the severity of OSAS, although it may have a clinically relevant effect. A beneficial impact on daytime sleepiness and on the clinical consequences OSAS has on daily activities has been observed. In addition, after classifying patients according to severity, a decrease in AHI and an improvement in parameters associated with lipid metabolism were observed in severe OSAS.

The minimum distance leading to a clinically important change is not well established in OSAS, especially when the subjects’ baseline situation is sedentary. In our program, the daily distance walked by the experimental group was 602 m longer than that of the control group. The average number of steps per day was similar to the 5388 average steps described in a meta-analysis involving subjects with OSAS and a walking program [22], ranging from the 7734 steps per day reported by Mendelson et al. [23] to the 1570 steps per day reported by Bamberga et al. [24]. Measuring steps using a pedometer may encourage all patients to increase their physical activity [25], which may diminish the effect of our intervention.

In the present study, it was observed that physical activity caused a decrease in body mass index, a result that has not been reported in previous studies [25,26,27]. Weight loss was observed in both groups. However, the decrease in AHI was higher in the intervention group. Therefore, there seems to be an issue linked to the exercise. Our patients were sedentary; in this population, the accumulation of fluid in the legs and its nocturnal redistribution in the neck can worsen OSAS. In this sense, the physical activity can individually act on a mechanism of disease for OSAS, which is the redistribution of fluid in the pharynx walls during sleep and improve OSAS [27]. Additionally, the loss of fat around the upper airway can increase the pharyngeal lumen [28].

A noteworthy feature of our study was its 24-week duration, which is longer than most clinical trials in which physical activity is the primary intervention of the rehabilitation program [12,25,26,27,29], typically ranging between 1 week [27] and 12 weeks [12,30]. This longer duration probably led to, in both groups, a decrease in body weight, neck circumference, and daytime sleepiness, and to a lower impact of daytime sleepiness on activities of daily living.

Several studies have examined the impact of healthy habits on daytime sleepiness [31,32,33]. The results of the present study show that daytime sleepiness decreased in the interventional group and can have a beneficial effect on quality of life and occupational health [2,3]. The impact of exercise on quality of life and daily activities in OSAS has been analyzed by other authors [12,34]. To our knowledge, this is the first clinical trial to demonstrate a positive effect during 6 months on different aspects of daily living using the Functional Outcomes of Sleep Questionnaire.

The main objective of the study was to determine the effectiveness of a progressive walking program in reducing the AHI. In our study in the control group, a decrease of one event/h was observed vs. six events/h in the experimental group. Therefore, it was possible to decrease the mean reached in a meta-analysis whose main objective was to determine the effect of exercise on AHI (−6.27 events/h) [35]. However, in the clinical trials included [12,25,26,27,31,36,37], the program was supervised and developed during hospital check-ups. In our research, AHI decreased by six events/h in the interventional group. Although the change was not statistically significant, this finding is relevant, as a 1-point increase in AHI in men with mild–moderate OSAS is associated with a 6% increase in stroke risk [38]. Our result is satisfying because untreated patients with severe OSAS have increased vascular morbidity and mortality [5,10,38]. Therefore, it is vital to decrease the number of respiratory events, as well as other vascular risk factors [39,40].

Besides, it should be noted that in the severe OSAS group, AHI decreased by 15 events/h, which is higher than average [35] and higher than that observed in various previous studies [12,26,27,41]. One advantage of our study is that the physical exercise program was home-based and easily performed by all patients, which allows us to generalize this recommendation to treat and prevent chronic conditions [42], as well as to reduce body weight and dyslipidemia, considered as cardiovascular risk factors [39,40,42,43].

### Limitations

Basal physical activity was determined by the YPAS questionnaire and allowed us to include sedentary subjects. Therefore, the results have to be limited to this population. Besides, because it was not possible to measure baseline physical activity with a pedometer, and we were not able to determine whether the program increased it. Nevertheless, significant differences were found in the number of daily steps in favor of the intervention group.

The diagnosis and severity of OSAS were determined in a single night, which may introduce additional variability [21]. Besides, participants randomly assigned to the control group also performed physical exercise, which may have decreased the magnitude of the effect of the program. It has been described that the use of a pedometer can motivate patients to increase physical activity [25] and that maintaining activity logs by the patients can lead to recall bias. However, this circumstance was common to both groups. On the other hand, this fact could increase the physical exercise in the control arm and decrease the effect of our intervention.

## 5. Conclusions

In sedentary patients with OSAS, physical exercise reduces body weight and sleepiness, and alleviates its effect on the patients’ perceived wellbeing. Furthermore, in patients with severe OSAS, the incorporation of a healthy habit has a positive and clinically relevant effect, reducing the number of respiratory disorders and improving the lipid profile.

## Figures and Tables

**Table 1:** Baseline characteristics of the subjects included in the study.

Anthropometric Data	Intervention Group (*n* = 34)	Control Group (*n* = 34)	*p*-Value
Age (years)	52 ± 6.6	50 ± 9.5	0.335
Men, *n* (%)	20 (59)	23 (67)	0.615
BMI (kg/m^2^)	32 ± 5.2	32 ± 4.3	0.527
Neck circumference (cm)	41 ± 3.5	40 ± 3.8	0.620
Waist-to-hip ratio	0.94 ± 0.529	0.94 ± 0.1	0.992
Physical Activity			
YPAS, Score	35 ± 10.6	34 ± 10.7	0.659
Questionnaires			
ESS, Score	9 ± 4.5	10 ± 4.3	0.370
FOSQ, Total Score	79 ± 19.3	77 ± 17.1	0.577
General Productivity Score	3.2 ± 0.82	3.1 ± 0.68	0.575
Activity Level Score	3.0 ± 0.84	3.0 ± 0.68	0.863
Vigilance Score	2.9 ± 1.01	3.0 ± 0.83	0.639
Social Outcomes Score	3.4 ± 1.04	3.4 ± 1.01	0.972
Intimacy and Sexual Relationships Score	3.2 ± 2.82	2.8 ± 1.27	0.166
Sleep-Disordered Breathing			
AHI	29 ± 19.7	27 ± 10.4	0.604
SpO_2_ mean (%)	94 ± 1.6	94 ± 1.7	0.677
T90 (%)	5.7 ± 11.64	3.6 ± 10.32	0.441
Oxygen desaturation index	33 ± 21.1	25 ± 10.7	0.032
Biochemical Parameters			
Urea (mg/dL)	35.7 ± 11.47	31.8 ± 6.47	0.093
Creatinine (mg/dL)	0.85 ± 0.286	0.80 ± 0.102	0.385
ALT (U/L)	28 ± 10.5	25 ± 10.5	0.252
AST (U/L)	23 ± 6.4	21 ± 5.0	0.272
Blood glucose (mg/dL)	104 ± 16.5	94 ± 14.8	0.014
Total Cholesterol, (mg/dL)	206 ± 31.6	204 ± 46.1	0.881
HDL-col, (mg/dL)	46 ± 11.4	48 ± 14.4	0.441
LDL-col, (mg/dL)	132 ± 28.4	137 ± 23.4	0.452
TGL, (mg/dL)	138 ± 69.1	132 ± 66.9	0.728

Values expressed with mean ± SD. BMI—Body Mass Index; YPAS—Yale Physical Activity Survey; ESS—Epworth Sleepiness Scale; FOSQ—Functional Outcomes of Sleep Questionnaire; AHI—Apnea–Hypopnea Index; SpO_2_—Peripheral capillary oxygen saturation measured by pulse oximetry; T90—Recording time spent with SpO_2_ <90%; ALT—Alanine aminotransferase; AST—Aspartate aminotransferase; HDL-col—High-density lipoprotein cholesterol; LDL—Low-density lipoprotein cholesterol; TGL—Triglycerides. The *p*-value represents the level of statistical significance of the Student *t*-test.

**Table 2:** Within-group changes in the intervention and control groups.

Variables	Intervention Group (*n* = 29)					Control Group (*n* = 29)				
	Baseline	After 6 Months	Mean Difference	95% IC	*p*-Value	Baseline	After 6 Months	Mean Difference	95% IC	*p*-Value
Anthropometric Data										
BMI (kg/m^2^)	32 ± 4.1	31 ± 4.1	0.759	0.087–1.431	0.028	32 ± 4.3	31 ± 4.5	0.653	0.195–1.111	0.007
Neck circumference (cm)	40.8 ± 3.61	39.8 ± 2.95	0.982	0.420–1.544	0.001	40.8 ± 3.	39.8 ± 2.92	0.845	0.373–1.316	0.001
Waist-to-hip ratio	0.94 ± 0.06	0.94 ± 0.052	0.007	−0.022–0.007	0.325	0.93 ± 0.08	0.93 ± 0.08	0.004	−0.009–0.016	0.543
Physical Activity										
Steps/day	-	4380 ± 3018			NA	-	3778 ± 2273			NA
Questionnaires										
ESS, Score	9.9 ± 4.42	7.7 ± 4.47	2.172	0.898–3.446	0.013	9.8 ± 4.56	9.2 ± 3.89	0.724	−0.529–1.977	0.292
FOSQ, Total Score	32 ± 4.1	31 ± 4.1	5.720	−10.407–1.034	0.028	32 ± 4.3	31 ± 4.5	0.086	−7.715–7.887	0.007
General Productivity Score	3.2 ± 0.70	3.4 ± 0.70	−0.196	−457–0.064	0.133	3.2 ± 0.63	3.1 ± 0.72	0.069	−0.151–0.289	0.526
Activity Level Score	3.0 ± 0.88	3.3 ± 0.54	−0.357	−0.639–(−0.075)	0.015	3.0 ± 0.71	3.2 ± 0.61	−0.189	−0.393–0.014	0.067
Vigilance Score	2.9 ± 1.05	3.4 ± 0.74	−0.500	0.854–(−0.146)	0.007	3.0 ± 0.89	3.2 ± 0.65	−0.234	−0.529−0.059	0.114
Social Outcomes Score	3.3 ± 1.12	3.5 ± 0.88	−0.214	−0.600–0.171	0.264	3.4 ± 1.08	3.4 ± 0.73	−0.034	−0.434–0.365	0.861
Intimacy and Sexual Relationships Score	3.2 ± 1.17	3.2 ± 1.25	0.071	−0.163–0.306	0.537	2.8 ± 1.28	3.1 ± 1.13	−0.241	−0.668–0.186	0.257
Sleep-Disordered Breathing										
AHI	29 ± 20.8	23 ± 13.1	6.062	−0.242–10.366	0.126	27 ± 9.9	25 ± 16.29	1.152	−4.915–7.218	0.778
SpO_2_ mean (%)	93 ± 1.7	93 ± 2.5	0.176	−0.565–0.916	0.630	94 ± 1.8	94 ± 1.9	−0.243	−0.974–0.487	0.501
T90 (%)	6 ± 6.4	6 ± 5.1	0.540	−3.829–4.909	0.802	4 ± 4.14	4 ± 4.2	0.033	−4.952–5.017	0.989
Oxygen desaturation index	33 ± 22.4	27 ± 18.9	6.091	−0.464–12.647	0.067	25 ± 11.0	25 ± 15.4	0.368	−5.587–6.323	0.900
Biochemical Parameters										
Urea (mg/dL)	37 ± 12.2	38 ± 12.7	−1.500	−4.491–1.491	0.313	32 ± 6.4	33 ± 8.6	−1.552	−4.783–1.680	0.334
Creatinine (mg/dL)	0.84 ± 0.31	0.86 ± 0.38	−0.017	−0.094–0.061	0.661	0.79 ± 0.09	0.79 ± 0.135	0.001	−0.030–0.031	0.982
ALT (U/L)	28 ± 11.1	25 ± 8.1	2.798	−1.173–6.745	0.160	25 ± 11.1	24 ± 10.6	0.966	−2.454–4.385	0.568
AST (U/L)	23 ± 6.5	21 ± 5.2	1.786	−1.084–4.655	0.213	20 ± 4.7	21 ± 7.5	−0.862	−3.771–2.047	0.549
Blood glucose (mg/dL)	103 ± 17.7	100 ± 33.6	3.250	−5.053–11.553	0.429	95 ± 15.8	95 ± 13.6	0.103	−3.958–4.165	0.959
Total cholesterol (mg/dL)	206 ± 32.1	194 ± 37.5	12.786	−0.246–25.817	0.054	203 ± 47.7	206 ± 36,1	−2.926	−23.206–17.353	0.770
HDL-col, (mg/dL)	46 ± 9.4	47 ± 9.5	−0.714	−3.912–2.483	0.650	48 ± 14.9	50 ± 12.3	−1.448	−5.245–2.348	0.441
LDL-col, (mg/dL)	132 ± 28.7	121 ± 33.6	10.643	−0.090–21.376	0.052	136 ± 22.7	129 ± 31.7	7.310	−2.183–16.804	0.126
TGL (mg/dL)	138 ± 74.2	134 ± 81	4.393	−24.381–33.166	0.756	132 ± 71.7	131 ± 57.1	1.034	−17.462–19.531	0.910

Values are expressed as the mean ±SD. IC—Interval Confidence; BMI—Body Mass Index; ESS—Epworth Sleepiness Scale; FOSQ—Functional Outcomes of Sleep Questionnaire; AHI—Apnea–Hypopnea Index; SpO_2_—Peripheral capillary oxygen saturation measured by pulse oximetry; T90%—recording time spent with SpO_2_ <90%; ALT—alanine aminotransferase; AST—aspartate aminotransferase; HDL-col—high-density lipoprotein cholesterol; LDL—low-density lipoprotein cholesterol; TGL—triglycerides; NA—not applicable. The *p*-value represents the level of statistical significance of the Student paired *t*-test.

**Table 3:** Impact of the physical exercise program analyzed by OSAS severity.

Variables	Moderate OSAS Group (*n* = 41)					Severe OSAS Group (*n* = 17)				
	Baseline	After 6 Months	Mean Difference	95% IC	*p*-Value	Baseline	After 6 Months	Mean Difference	95% IC	*p*-Value
Anthropometric Data										
BMI (kg/m^2^)	32 ± 3.8	31 ± 4.1	0.530	0.055–1.005	0.030	32 ± 5.0	30 ± 4.7	1.117	0.393–1.841	0.005
Neck circumference (cm)	40 ± 3.2	39 ± 3.1	0.850	0.412–1.289	0.001	41 ± 4.6	40 ± 3.7	1.058	0.404–1.713	0.003
Waist-to-hip ratio	0.93 ± 0.07	093 ± 0.067	−0.002	−0.011–0.007	0.632	0.94 ± 0.064	0.94 ± 0.062	0.000	−0.024–0.024	1.000
Physical Activity										
Steps/day	-	4261 ± 2026.3			NA	-	3633 ± 2462.8			NA
Questionnaires										
ESS, Score	9.7 ± 4.43	8.5 ± 3.95	1.561	0.601–2.521	0.012	8.7 ± 4.61	8.3 ± 4.95	1.176	−0.961–3.314	0.587
FOSQ, Total Score	76 ±18.0	81 ± 15.0	−4.434	−8.753–(−0.114)	0.044	81 ± 20.4	80 ± 23.4	1.159	−10.841–13.158	0.840
Sleep-Disordered Breathing										
AHI	20 ± 4.76	23 ± 15.7	1.683	−5.660–2.294	0.228	45 ± 20.6	30 ± 26.3	14.659	7.356–21.961	0.002
SpO_2_ mean (%)	94 ± 1.6	94 ± 2.2	0.148	−0.449–0.745	0.620	93 ± 1.9	93 ± 2.3	−0.471	−1.501–0.560	0.347
T90 (%)	5 ± 6.2	4 ± 4.3	0.285	−4.092–4.663	0.896	6 ± 5.9	6 ± 4.9	0.288	−3.374–3.950	0.870
Oxygen desaturation index	24 ± 9.7	23 ± 14.7	0.842	−4.900–5.873	0.737	43 ± 25.1	34 ± 20.2	8.988	0.182–17.794	0.046
Biochemical Parameters										
Urea (mg/dL)	35 ± 30.7	36 ± 12.0	−1.900	−4.24–0.824	0.166	33 ± 7.9	34 ± 8.2	−0.647	−4.237–2.943	0.707
Creatinine (mg/dL)	0.81 ± 0.27	0.80 ± 0.293	0.007	−0.025–0.040	0.645	0.84 ± 0.866	0.88 ± 0.258	−0.044	−0.162–0.073	0.432
ALT (U/L)	26 ± 10.5	25 ± 10.0	0.500	−2.554–3.554	0.742	27 ± 12.5	22 ± 7.6	5.059	0.387–9.731	0.036
AST (U/L)	22 ± 6.2	22 ± 6.9	0.000	−2.673–2.673	1.000	21 ± 4.7	20 ± 5.1	1.471	−1.305–4.246	0.278
Blood glucose (mg/dL)	100 ± 18.5	98.4 ± 29.1	1.875	−4.398–8.148	0.549	96 ± 13.8	95 ± 14.0	1.117	−2.,287–4.523	0.497
Total Cholesterol (mg/dL)	201 ± 42.4	200 ± 40.8	1.104	−14.763–16.970	0.889	214 ± 33.4	201 ± 26.7	13.471	−2.678–29.620	0.096
HDL-col, (mg/dL)	49 ± 14.3	48 ± 11.4	0.250	−2.924–3.424	0.874	45 ± 7.8	49 ± 10.3	−4.235	−7.253–(−1.218)	0.009
LDL-col, (mg/dL)	131 ± 24.2	124 ± 36.2	6.950	−1.596–15.496	0.108	141 ± 28,6	127 ± 22.6	13.647	0.856–26.438	0.038
TGL (mg/dL)	134 ± 67	139 ± 75.7	−5.650	−24.758–13.458	0.553	37 ± 84.4	115 ± 48.7	22.294	−10.853–55.441	0.173

Values are expressed as the mean (SD). BMI—Body Mass Index; ESS—Epworth Sleepiness Scale; FOSQ—Functional Outcomes of Sleep Questionnaire; AHI—Apnea–Hypopnea Index; T90—recording time spent with SpO_2_ <90%; ALT—alanine aminotransferase; AST—aspartate aminotransferase; HDL-col—high-density lipoprotein cholesterol; LDL—low-density lipoprotein cholesterol; TGL—triglycerides; NA—not applicable. The *p*-value represents the level of statistical significance of the Student paired *t*-test for Moderate OSAS and Wilcoxon’s signed-rank test for Severe OSAS.
